# Analysis of the correlation between neutrophil percentage-to-albumin ratio, neutrophil-to-lymphocyte ratio and platelet-to-lymphocyte ratio with short-term prognosis in acute ischemic stroke patients undergoing intravenous thrombolysis

**DOI:** 10.3389/fneur.2025.1512355

**Published:** 2025-04-14

**Authors:** Congcong Deng, Bingyi Liu, Mengmeng Wang, Chenlu Zhu, Yingtao Xu, Jiehui Li, Ying Bai

**Affiliations:** ^1^Department of Neurology, Dalian University Affiliated Xinhua Hospital, Dalian, Liaoning, China; ^2^Graduate School, Dalian University, Dalian, Liaoning, China

**Keywords:** acute ischemic stroke, outcome, neutrophil percentage-to-albumin ratio, neutrophil-to-lymphocyte ratio, platelet-to-lymphocyte ratio

## Abstract

**Introduction:**

The purpose of this study was to evaluate the association between four easy-to-measure inflammatory markers and the 90-day outcomes with acute ischemic stroke (AIS) patients who received intravenous thrombolytic therapy with recombinant tissue plasminogen activator (rt-PA). These included the neutrophil percentage-to-albumin ratio (NPAR), the neutrophil-to-lymphocyte ratio (NLR), the platelet-to-lymphocyte ratio (PLR), and the combined NPAR+NLR index.

**Methods:**

This study enrolled 151 AIS patients who were treated with rt-PA (0.9 mg/kg). Clinical data were collected and NPAR, NLR, PLR were calculated from the admission blood work. The patients were followed up for 90 days after stroke onset and subsequently categorized into two groups based on the modified Rankin Scale (mRS): a favorable outcome group (111 patients, mRS ≤ 2) and a poor outcome group (40 patients, mRS > 2).

**Results:**

In this study, we foud elevated level of albumin and lymphocyte counts are protective factors for short-term prognosis. Age, neutrophil percentage, NPAR, NLR, PLR, NIHSS score, and fasting blood glucose (FBG) are associated with poor short-term prognosis. Among these, age, NPAR, NLR, and NIHSS score are independent risk factors for poor short-term prognosis.

**Discussion:**

NPAR, NLR, PLR, and the combined NPAR+NLR index may have predictive value for poor short-term outcomes in AIS patients following thrombolysis. NPAR demonstrates the highest predictive capability, in the following order: NPAR > NPAR+NLR > NLR > PLR.

## 1 Introduction

AIS is a prevalent neurological disorder among the elderly, with high rates of incidence, mortality, and disability. For AIS patients presenting within 4.5 h of symptom onset, intravenous thrombolytic therapy (IVT) using rt-PA is considered the optimal treatment. This intervention is effective in salvaging the ischemic penumbra and restoring cerebral blood flow, thereby improving patient outcomes (1). However, despite stringent adherence to IVT indications and contraindications, some patients still experience adverse outcomes.

Inflammation and oxidative stress are critical factors influencing reperfusion prognosis in AIS patients (2). During the acute phase of AIS, there is a substantial release of pro-inflammatory cytokines and damage-associated molecular patterns (DAMPs), leading to the recruitment and activation of neutrophils. Neutrophils, which are among the first inflammatory cells to accumulate in the cerebral microvasculature, release reactive oxygen species (ROS), which are major contributors to reperfusion injury following IVT (3–5). Neutrophils serve as markers of both acute and chronic inflammation, promoting inflammation (6, 7) and thrombosis (7). Additionally, neutrophils are implicated in the pathogenesis of atherosclerosis (8) and may contribute to recurrent arterial occlusive events.

Serum albumin plays a crucial role in scavenging ROS (9) and may exert anti-inflammatory effects by inhibiting neutrophil dispersion (10), thus offering neuroprotection and potentially reducing the risk of recurrent ischemic stroke (RIS), thereby improving patient prognosis. Conversely, low serum albumin levels indicate poor nutritional status and inflammation (11). The NPAR represents a novel inflammatory biomarker. Compared to single factors, NPAR provides a more comprehensive reflection of systemic immune and inflammatory responses. However, the relationship between NPAR and outcomes in AIS patients undergoing thrombolysis remains incompletely understood.

Lymphocytes play a vital role in tissue repair and inflammation control due to their involvement in various inflammatory pathways (12). A low NLR at admission may serve as a valuable marker for predicting neurological recovery 90 days after a stroke (13). Moreover, admission NLR could be a significant biomarker for predicting outcomes in patients with large vessel occlusive stroke (14).

Platelets are crucial for both thrombosis and inflammation (15). During AIS, platelets are rapidly activated, adhering to damaged endothelial surfaces and releasing inflammatory factors that recruit additional inflammatory cells to the injury site and amplify the inflammatory response (16). In contrast, acute stress typically reduces lymphocyte counts, which are essential for coordinating healing and controlling the spread of inflammation (12). Recent evidence suggests that a higher PLR is associated with increased rates of recurrent stroke, mortality, and poor functional outcomes following stroke. PLR has emerged as a potential novel biomarker in IVT for AIS, showing promise in predicting functional outcomes (17, 18).

This study aims to evaluate the predictive value of NPAR, NLR, PLR, and the combined NPAR+NLR index for the 90-day prognosis of AIS patients receiving rt-PA IVT, to help clinicians more effectively identify high-risk patients with poor outcomes.

## 2 Materials and methods

### 2.1 Patients

This study was approved by the Ethics Committee of Xinhua Hospital, affiliated with Dalian University (No. 2023–151-01). A single-center, retrospective study was conducted, involving 168 AIS patients treated at the Neurology Department of Xinhua Hospital between May 2017 and November 2023. The inclusion criteria were as follows: (1) a new AIS diagnosis based on clinical symptoms and neuroimaging, with intracranial hemorrhage excluded by CT; (2) presentation within 4.5 h of symptom onset and treatment with IVT using rt-PA (0.9 mg/kg), with a maximum dose of 90 mg; (3) age ≥ 18 years. The exclusion criteria included: (1) intracranial hemorrhage or a history of intracranial hemorrhage; (2) intra-arterial bridging therapy, mechanical thrombectomy, or thrombus aspiration following thrombolysis; (3) a history of stroke with a pre-stroke mRS score > 1; (4) significant trauma, surgery, or burns within 90 days before symptom onset; (5) acute or chronic infection, or use of anti-infective drugs, steroids, or immunosuppressants; (6) systemic diseases such as severe liver or renal dysfunction, malignancy, acute myocardial infarction, or hematological disorders; (7) other severe conditions within 90 days post-thrombolysis that could affect the mRS score.

### 2.2 Definitions

Neurological function was assessed using the mRS 90 days after thrombolysis. A score of ≤2 was considered indicative of a good prognosis, while a score > 2 indicated a poor prognosis. Trained neurologists conducted face-to-face or telephone interviews with patients and their families 90 days after discharge to determine the mRS score. Thrombolysis was administered according to American guidelines for early management of AIS and the Chinese Stroke Guidelines (19, 20). The rt-PA dose was 0.9 mg/kg (maximum 90 mg), prepared as a 1 mg/mL solution. Ten percent of the total dose was administered intravenously as a bolus over 1 min, followed by the remaining 90% as a continuous infusion over 1 h.

### 2.3 Specimen collection and detection method

Blood samples for routine laboratory tests were collected before any treatment (pre-thrombolysis, within 4.5 h of symptom onset). Complete blood counts were analyzed using the Sysmex XN-B3 automated hematology analyzer (Japan), and liver function tests were conducted using the Hitachi 7,600–020 automated biochemical analyzer (Japan).

### 2.4 Statistical analysis

Statistical analyses were conducted using Excel and SPSS 27.0 software. The normality of continuous variables was assessed with the Shapiro–Wilk test. Normally distributed data were expressed as mean ± standard deviation (SD) and compared between groups using the independent samples *t*-test. Non-normally distributed data were presented as median and interquartile range (IQR) and compared using the Mann–Whitney U test. Categorical data were described with frequency and percentage (n, %) and compared using the χ^2^ test. Baseline characteristics were compared between the good and poor prognosis groups. Variables with significant differences were included in univariate logistic regression analysis. Significant variables from univariate analysis were then incorporated into multivariate logistic regression analysis to identify independent risk factors for poor prognosis post-thrombolysis. The diagnostic value of each predictive factor was evaluated using ROC curves, with accuracy assessed by the AUC, sensitivity, and specificity. Statistical significance was set at *p* < 0.05.

## 3 Results

### 3.1 Study population

**Figure 1:**
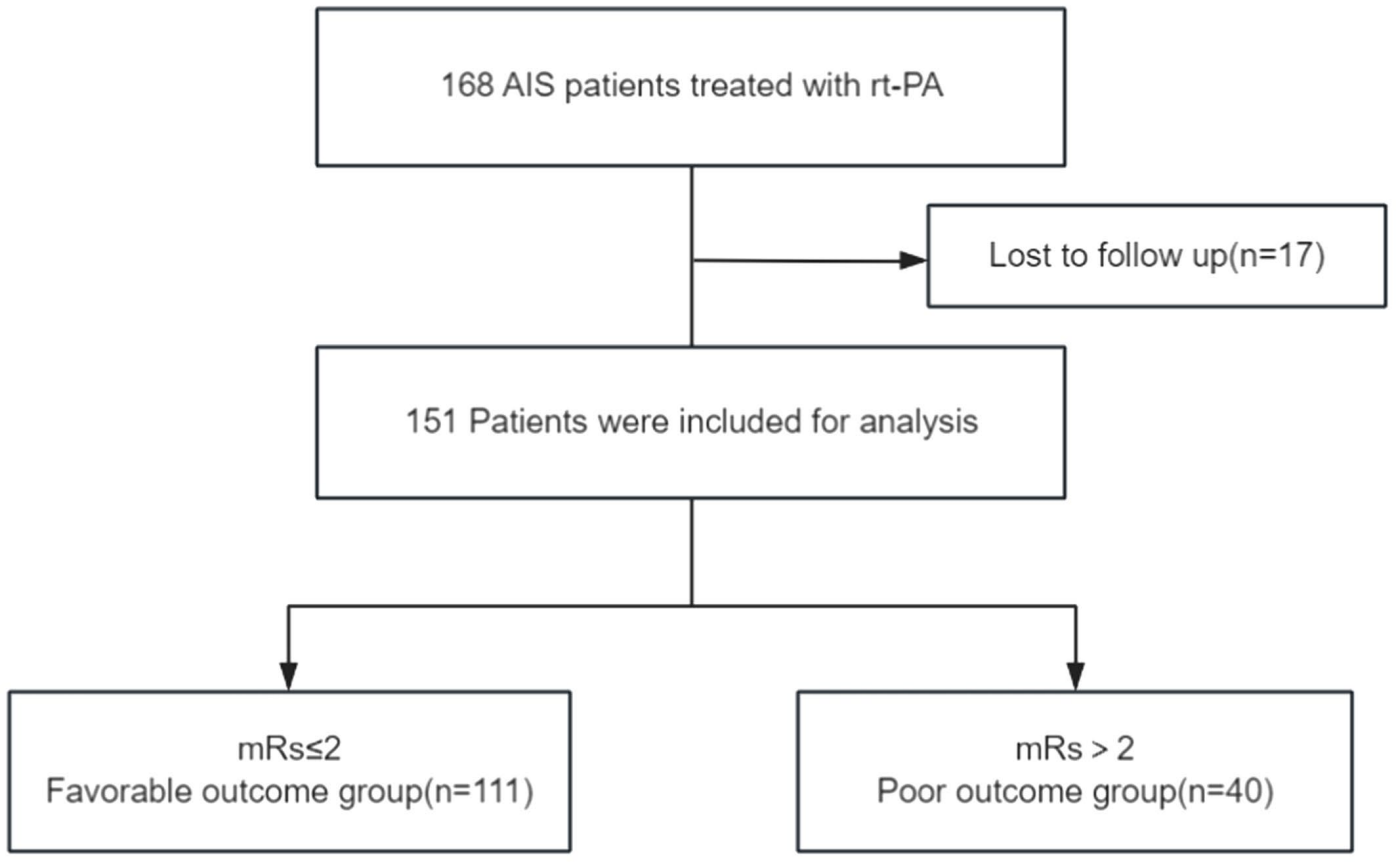
Flow-chart describing the selection of the patients for the study.

**Figure 2:**
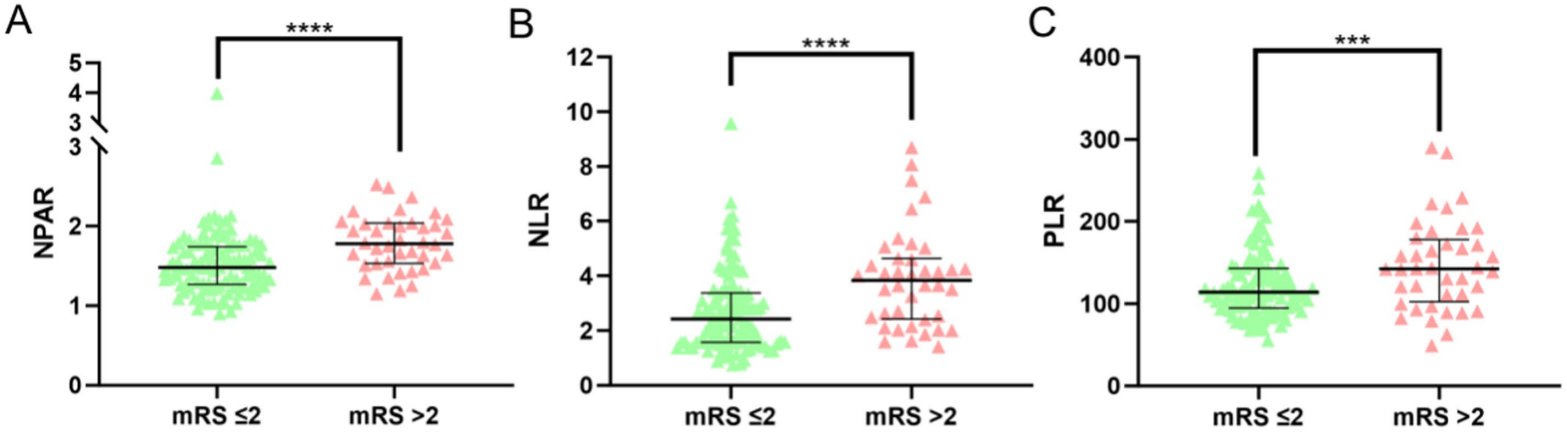
NPAR, NLR, PLR levels and distributions in favorable outcome and poor outcome groups. (Statistical significance is denoted by asterisks: ***p < 0.05, ****p < 0.001).

A total of 168 AIS patients who underwent IVT were initially included in this study. After applying the inclusion and exclusion criteria, 17 patients were excluded due to missing or incomplete follow-up data, resulting in a final cohort of 151 AIS patients. Of these, 102 were male (67.5%) and 49 were female (32.5%; Figure 1). The favorable prognosis group, defined as having an mRS score ≤ 2, consisted of 111 patients (77 male, 34 female) with a mean age of 66.08 ± 10.66 years. The poor prognosis group, defined as having an mRS score > 2, comprised 40 patients (25 male, 15 female) with a mean age of 74.28 ± 8.84 years. A comparison of baseline characteristics between the two groups revealed no significant differences in gender, history of stroke, hypertension, type 2 diabetes, coronary heart disease, smoking, alcohol consumption, platelet count, or the duration from symptom onset (*p* > 0.05). However, the poor outcome group exhibited significantly higher values in age, neutrophil percentage, absolute neutrophil count, NIHSS score, FBG levels, NPAR, NLR, PLR (Figure 2) compared to the favorable outcome group (*p* < 0.05). Additionally, albumin and lymphocyte counts were significantly lower in the poor outcome group (*p* < 0.05). Refer to Table 1 for a detailed comparison.

**Table 1:** Sociodemographic and clinical characteristics of the patients.

Variable	Favorable outcome (*n* = 111)	Poor outcome (*n* = 40)	t/z/χ^2^	*p*-value
Male, n (%)	77 (69.4)	25 (62.5)	χ^2^ = 0.633	0.426
Age in years ( $\bar{X}$ ±s)	66.08 ± 10.66	74.28 ± 8.84	*t* = −4.350	<0.001
Stoke history, n (%)	14 (12.6)	10 (25.0)	χ^2^ = 3.375	0.066
Hypertension, n (%)	62 (41.1)	26 (65.0)	χ^2^ = 1.011	0.315
Type 2 diabetes, n (%)	30 (27.0)	17 (42.5)	χ^2^ = 3.284	0.070
CHD, n (%)	17 (15.3)	7 (17.5)	χ^2^ = 0.105	0.746
Smoking, n (%)	30 (27.0)	10 (25.0)	χ^2^ = 0.062	0.803
Drinking, n (%)	22 (19.8)	5 (12.5)	χ^2^ = 1.073	0.300
NE%	63.14 ± 10.89	70.38 ± 10.51	*t* = −3.639	<0.001
NE in ×10^9^/L	4.19 (3.15,5.93)	5.28 (4.09,6.20)	z = −2.231	0.026
A	42.00 (39.10,45.60)	40.20 (38.00,42.90)	z = −2.779	0.005
LY in ×10^9^/L	1.92 (1.52,2.40)	1.45 (1.18,1.65)	z = −3.646	<0.001
PLT in ×10^9^/L	211.00 (180.00,266.00)	193.00 (166.25,244.00)	z = −1.693	0.090
NPAR	1.48 (1.27,1.74)	1.78 (1.53,2.04)	z = −4.179	<0.001
NLR	2.42 (1.57,3.37)	3.83 (2.42,4.63)	z = −3.955	<0.001
PLR	113.92 (94.68,142.86)	142.14 (102.49,177.99)	z = −2.690	0.007
NIHSS	4.00 (7.00,14.75)	9.50 (7.00,14.75)	z = −6.66	<0.001
FBG, in mmol/L	6.80 (5.60,8.50)	7.80 (6.43,9.70)	z = −3.212	0.001
Time onset-to-sample	185.00 (135.00,220.00)	172.00 (142.00,209.25)	z = −0.565	0.572

CHD, coronary heart disease; NE, neutrophil; A, albumin; LY, lymphocyte; PLT, platelet; NPAR, neutrophil percentage to albumin ratio; NLR, neutrophil to lymphocyte ratio; PLR, platelet to lymphocyte ratio; NIHSS, National Institute of Health Stroke Scale; FBG, fasting blood glucose.

### 3.2 Association between NPAR, NLR, PLR and poor outcome

Univariate logistic regression analysis revealed that gender, history of stroke, hypertension, type 2 diabetes, coronary artery disease, smoking, alcohol consumption, neutrophil count, platelet count, and the duration from symptom onset were not significantly associated with 90-day outcomes (*p* > 0.05). In contrast, age, neutrophil percentage, NPAR, NLR, PLR, NIHSS score, and FBG levels were identified as significant risk factors for poor outcomes in AIS patients receiving intravenous thrombolysis (*p* < 0.05). Conversely, elevated albumin levels and high lymphocyte counts were protective factors for short-term prognosis (*p* < 0.05; refer to Table 2).

**Table 2:** Univariate logistic regression analysis of short-term prognosis.

Variable	B	S.E	Wald	OR	*p*-value	95%CI	
						Lower	Upper
Male	−0.307	0.386	0.631	0.736	0.427	0.345	1.568
Age in years	0.082	0.021	15.262	1.085	<0.001	1.042	1.131
Stoke history	−0.837	0.464	3.258	0.433	0.071	0.174	1.08
Hypertension	−0.734	0.402	3.328	0.480	0.068	0.218	1.056
Type 2 diabetes	−0.691	0.385	3.226	0.501	0.072	0.236	1.065
CHD	−0.159	0.493	0.105	0.853	0.746	0.325	2.239
Smoking	0.105	0.423	0.062	1.111	0.803	0.485	2.546
Drinking	0.548	0.534	1.054	1.730	0.305	0.607	4.929
NE%	0.063	0.019	11.309	1.065	0.001	1.027	1.105
NE	0.163	0.085	3.675	1.177	0.055	0.996	1.390
A	−0.123	0.045	7.413	0.884	0.006	0.809	0.966
LY	−1.035	0.352	8.659	0.355	0.003	0.178	0.708
PLT	−0.005	0.004	2.219	0.995	0.145	0.988	1.002
NPAR	1.724	0.543	10.076	5.606	0.002	1.934	16.251
NLR	0.413	0.117	12.471	1.512	<0.001	1.202	1.902
PLR	0.011	0.004	7.677	1.011	0.006	1.003	1.020
NIHSS	0.313	0.057	30.267	1.367	<0.001	1.223	1.528
FBG	0.142	0.057	6.196	1.152	0.013	1.031	1.288
Time onset-to-sample	−0.002	0.003	0.325	0.998	0.569	0.992	1.005

CHD, coronary heart disease; NE, neutrophil; A, albumin; LY, lymphocyte; PLT, platelet; NPAR, neutrophil percentage-to-albumin-ratio; NLR, neutrophil-to-lymphocyte-ratio; PLR, platelet-to-lymphocyte-ratio; NIHSS, National Institute of Health Stroke Scale; FBG, fasting blood glucose.

Multivariate logistic regression analysis, adjusting for confounding factors such as neutrophil percentage, albumin, lymphocyte count, PLR, and FBG levels, revealed that age (OR = 1.116, 95% CI: 1.047–1.190, *p* < 0.05), NPAR (OR = 3.898, 95% CI: 1.079–14.087, *p* < 0.05), NLR (OR = 1.672, 95% CI: 1.056–2.647, *p* < 0.05), and NIHSS score (OR = 1.499, 95% CI: 1.280–1.756, *p* < 0.05) were independent risk factors for poor short-term prognosis (*p* < 0.05; refer to Table 3).

**Table 3:** Multivariate logistic regression analysis of short-term prognosis.

Variable	B	S.E	Wald	OR	*p*-value	95%CI	
						Lower	Upper
Age in years	0.110	0.033	11.421	1.116	0.001	1.047	1.190
NPAR	1.361	0.655	4.308	3.898	0.038	1.079	14.087
NLR	0.514	0.234	4.810	1.672	0.028	1.056	2.647
NIHSS	0.405	0.081	25.134	1.499	<0.001	1.280	1.756

NPAR, neutrophil percentage-to-albumin-ratio; NLR, neutrophil-to-lymphocyte-ratio; NIHSS, National Institute of Health Stroke Scale.

### 3.3 ROC curve analysis for short-term prognosis

**Figure 3:**
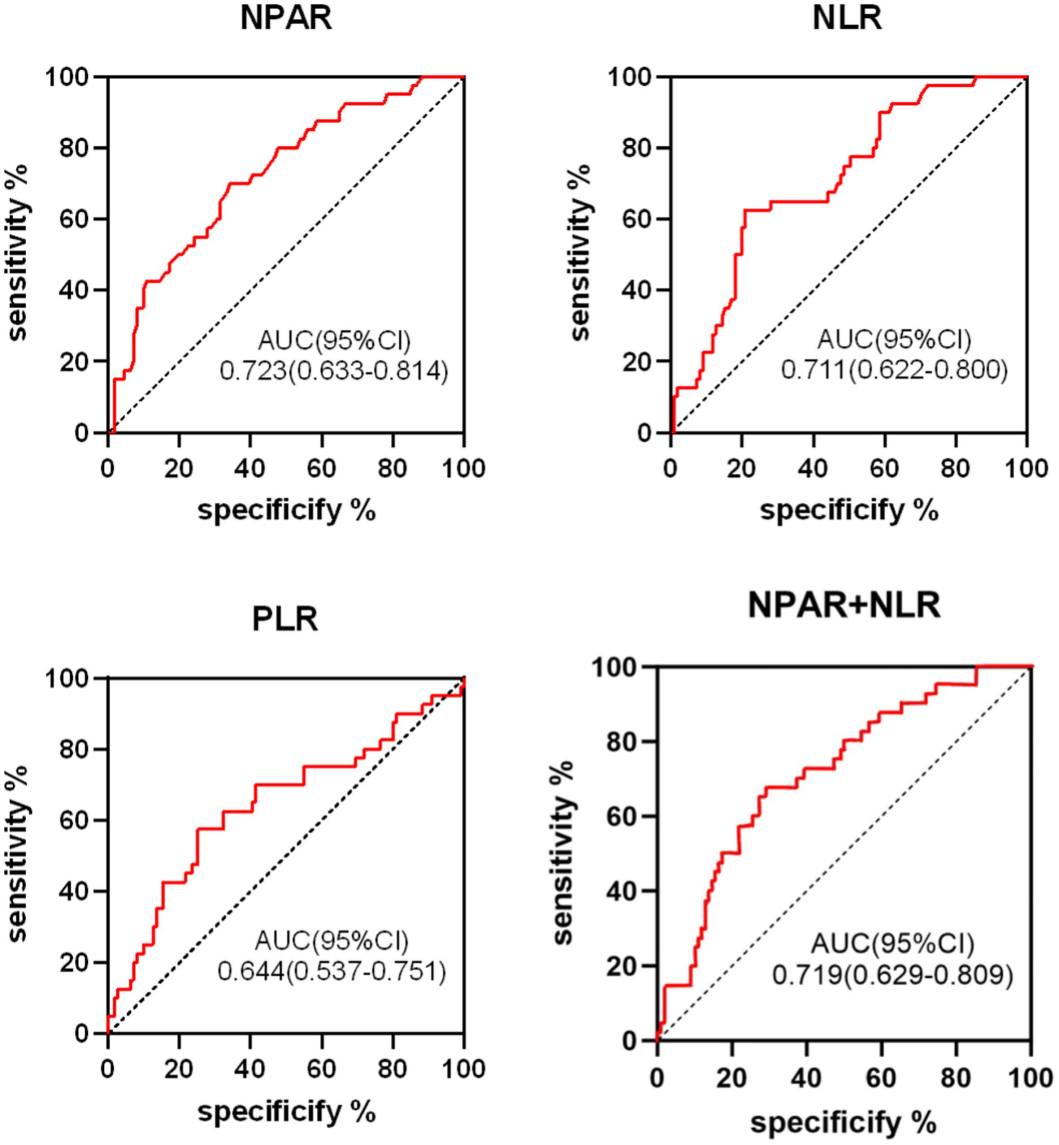
ROC curve analysis of NPAR, NLR, PLR, NPAR+NLR in predicting poor short-term outcomes in AIS patients following thrombolysis.

The predictive value of NPAR, NLR, PLR, and their combination (NPAR + NLR) for 90-day post-thrombolysis outcomes in AIS patients was assessed using ROC curves (Figure 3). The ROC analysis revealed that NPAR exhibited the highest predictive accuracy for adverse outcomes, with an AUC of 0.723 (95% CI: 0.633–0.814, *p* < 0.05). The NLR, with an AUC of 0.711 (95% CI: 0.622–0.800, *p* < 0.05), had an optimal threshold of 3.495, with a sensitivity of 62.5% and a specificity of 79.3%. For the combined predictor (NPAR + NLR), the ROC curve yielded an AUC of 0.719 (95% CI: 0.629–0.809, *p* < 0.05), with a sensitivity of 67.5% and a specificity of 71.2%. Detailed data are provided in Table 4.

**Table 4:** ROC curve for short-term prognosis.

Variable	AUC	*p*-value	the best cutoff value	95%CI	Sensitivity (%)	Specificity (%)
NPAR	0.723	0.000	1.615	0.633–0.814	70.0	65.8
NLR	0.711	0.000	3.495	0.622–0.800	62.5	79.3
PLR	0.644	0.007	138.34	0.537–0.751	57.5	74.8
NPAR+NLR	0.719	0.000	–	0.629–0.809	67.5	71.2

AUC, area under curve; NPAR, neutrophil percentage-to-albumin-ratio; NLR, neutrophil-to-lymphocyte-ratio; PLR, platelet-to-lymphocyte-ratio.

The ROC curve analysis indicates that NPAR offers superior predictive sensitivity, whereas NLR provides greater specificity. AUC values ranging from 0.5 to 0.7 are considered to have low diagnostic value, whereas values between 0.7 and 0.9 are indicative of moderate diagnostic value. Accordingly, levels of NPAR, NLR, PLR, and the combined NPAR + NLR exhibit notable prognostic values for short-term adverse outcomes. Specifically, NPAR exhibits the highest predictive efficacy, followed by the combined NPAR + NLR, NLR, and PLR in descending order of predictive value.

## 4 Discussion

This study included 151 patients who underwent intravenous thrombolysis for AIS, among whom 40 experienced adverse outcomes, corresponding to an incidence rate of 26.5%. Enhancing the efficacy of intravenous thrombolysis for a greater number of AIS patients, reducing disability and mortality rates, and improving the prediction of treatment outcomes are critical public health concerns. This study aimed to evaluate the prognostic value of routine blood and liver function biomarkers in predicting short-term outcomes after intravenous thrombolysis in AIS patients. This research seeks to provide clinicians with more objective, convenient, and efficient indicators for assessing prognosis.

Neutrophils are among the first white blood cells to infiltrate ischemic regions, breaching the blood–brain barrier within 6 h of AIS onset and peaking in cerebral infiltration between 24 and 48 h (21). These cells secrete various factors, including reactive oxygen species (ROS), proteases, cytokines, and chemokines (22), which contribute to neuronal cell death and exacerbate reperfusion injury. Post-stroke neutrophil-driven inflammation is associated with increased infarct volume, hemorrhagic transformation, stroke severity, and worse functional outcomes (23, 24). Clinically, stroke patients are more susceptible to infections, which may result in poorer functional outcomes. Regardless of the severity of infection, leukocyte levels typically rise post-stroke. To control for potential confounding factors, patients showing signs of infection were excluded from this study. Our findings are consistent with prior research, which shows higher neutrophil levels in the adverse outcome group compared to the favorable outcome group.

Albumin reflects both the body’s inflammatory and nutritional status. Low albumin levels are associated with cytokine activation and chronic inflammation (25). Serum albumin not only acts as a specific inhibitor of endothelial cell apoptosis (26), but also has significant antioxidant properties (27). Additionally, albumin may reduce post-AIS inflammatory responses by inhibiting neutrophil-endothelial adhesion (28). Malnutrition is a well-known risk factor for poor outcomes following stroke (29). Hypoalbuminemia in stroke patients may arise from malnutrition and/or underlying disease processes, such as renal or hepatic failure or malignancies. To ensure patient homogeneity, individuals with severe liver disease, renal failure, or malignancies were excluded. Our study found that serum albumin levels were lower in the adverse outcome group compared to the favorable outcome group. Univariate logistic regression analysis indicated that albumin (OR: 0.884, *p* = 0.006) is a protective factor for short-term prognosis following intravenous thrombolysis in AIS patients, which is consistent with previous literature.

NPAR, a novel composite biomarker for inflammation and oxidative stress, is considered an accurate reflection of inflammatory status and disease prognosis. A recent retrospective study involving 829 AIS patients demonstrated that NPAR is independently associated with the risk of stroke recurrence within 3 months, suggesting it may be a more effective biomarker for predicting stroke recurrence (30). Moreover, elevated NPAR can predict stroke-associated infections (SAI), and compared to albumin, neutrophil percentage, and NLR, NPAR may serve as a more effective biomarker for predicting SAI (31). While existing studies have confirmed that neutrophils and albumin are associated with functional outcomes in AIS patients undergoing reperfusion therapy (32, 33), there is insufficient evidence to suggest that NPAR predicts outcomes in AIS patients receiving intravenous thrombolysis. Our study reveals a positive correlation between NPAR and adverse functional outcomes. Multivariate logistic regression analysis, adjusting for confounding factors, identified NPAR (OR: 3.898, *p* = 0.038) as an independent risk factor for short-term adverse outcomes following thrombolysis. ROC curve analysis indicates that NPAR (AUC = 0.723, 95% CI: 0.633–0.814, *p* < 0.05) demonstrates higher predictive value for adverse outcomes compared to NPAR + NLR, NLR, and PLR, reflecting greater sensitivity and reliability in predicting poor outcomes in AIS patients. The combined NPAR + NLR indices demonstrated lower predictive value compared to NPAR alone, possibly due to overlapping information from the neutrophil count and sample size limitations. Further research with larger sample sizes is necessary to validate these findings.

Post-ischemic stroke lymphocyte count reduction indicates ongoing brain injury, heightened stress responses, and increased infection risk (34), correlating with poorer outcomes (35). Lymphocytes play a crucial role in anti-inflammatory and endothelial protective mechanisms, and their apoptosis is exacerbated by worsening atherosclerosis (36). To control for the impact of lymphocyte count influenced by systemic inflammation, patients with active infections, malignancies, and chronic diseases were excluded. Our study found a correlation between reduced lymphocyte counts and adverse outcomes. Univariate logistic regression analysis yielded an OR of 0.355, reinforcing the notion that lymphocyte count is a significant protective factor for prognosis in AIS patients undergoing intravenous thrombolysis, consistent with existing literature.

NLR is a reliable indicator of systemic inflammation and disease prognosis, and is widely used in various disease studies. In AIS patients, NLR correlates with stroke severity, short-term functional outcomes, and infarct recurrence (37). Additionally, NLR serves as an independent predictor of 3-month mortality in AIS patients (38). A prospective observational study of 341 ischemic stroke patients revealed that, after adjusting for initial NIHSS scores, both diabetes and NLR were independently associated with stroke progression, with NLR predicting adverse functional outcomes (39). Other research highlights that elevated NLR, measured 24–36 h post-thrombolysis, significantly predicts stroke-associated pneumonia and can forecast short- and long-term adverse outcomes, hemorrhagic transformation, and 1-year mortality (40). In our study, univariate logistic regression analysis indicated that NLR levels (OR: 1.512, *p* < 0.001) were significantly higher in the adverse outcome group compared to the favorable outcome group. Multivariate logistic regression identified NLR (OR = 1.672, *p* = 0.025) as an independent predictor of short-term adverse outcomes following thrombolysis. ROC curve analysis yielded an AUC of 0.711 (95% CI: 0.622–0.800, *p* < 0.05), demonstrating moderate predictive value with higher specificity.

In the context of AIS, thrombosis and vascular occlusion are primarily driven by the abnormal activation and excessive aggregation of platelets (41). Circulating platelets are critical effectors in the onset, progression, and resolution of stroke, exerting direct effects on endothelial cells, and their interactions with leukocytes can exacerbate inflammatory and thrombotic conditions. While rt-PA facilitates the conversion of plasminogen to plasmin, it also increases platelet activation and aggregation (42). Following thrombolysis, an increase in platelet count may trigger delayed thrombotic events, potentially leading to re-occlusion and recurrent thrombosis (43). Previous studies have suggested that elevated platelet counts increase the risk of ischemic stroke, but are not significantly associated with adverse outcomes (44). In our study, platelet counts in the adverse outcome group were not significantly higher than those in the favorable outcome group.

PLR serves as a marker of atherosclerotic inflammation, offering insights into the mechanisms underlying AIS, including hemostasis, thrombosis, and inflammatory pathways. Elevated PLR levels are potentially linked to the severity of coronary atherosclerosis and symptomatic carotid artery stenosis (45, 46). Additionally, high PLR can serve as an indirect marker for estimating infarct size, post-thrombectomy recanalization rates, and adverse outcomes (47). PLR has been shown to predict the likelihood of hemorrhagic transformation in younger AIS patients, highlighting its clinical significance in preventing such transformations (48). In patients undergoing mechanical thrombectomy, admission PLR may predict 3-month prognosis and mortality, with lower PLR levels associated with better clinical outcomes at 3 months (49). An observational study of patients receiving rt-PA treatment found that admission PLR before thrombolysis was associated with early neurological improvement and deterioration (22). Our study’s univariate logistic regression analysis indicated that the PLR level in the adverse outcome group (OR: 1.011, *p* = 0.006) was higher compared to the favorable outcome group, and PLR demonstrated predictive value for prognosis in AIS patients undergoing intravenous thrombolysis (AUC = 0.644, 95% CI: 0.537–0.751, *p* < 0.05).

Additionally, our study found that the poor outcome group had higher values for age (OR: 1.085, *p* < 0.001), NIHSS score (OR: 1.367, *p* < 0.001), and FBG level (OR: 1.152, *p* = 0.013) compared to the favorable outcome group. Age (OR = 1.116, 95% CI: 1.047–1.190, *p* < 0.05) and NIHSS score (OR = 1.499, 95% CI: 1.280–1.756, *p* < 0.05) were also identified as independent risk factors for short-term adverse outcomes, consistent with traditional predictors found in previous research.

### 4.1 Limitations

As indicators that can be applied even in underdeveloped medical settings, NPAR, NLR, and PLR are simple, cost-effective, and readily available. Composite indices are more stable than single markers, which may be influenced by various physiological and pathological conditions. Importantly, these composite indicators enhance the prognostic value of individual markers by integrating various mechanisms to predict outcomes, as demonstrated by ROC curve analysis. A notable advantage of our study is the use of baseline data from pre-thrombolysis blood samples, offering an ideal context for evaluating the impact of these indicators on outcomes. However, our study has limitations: it is a small-sample, single-center retrospective observational study, which may introduce selection bias and limit generalizability. Additionally, dynamic monitoring of NPAR, NLR, and PLR levels was not conducted, and their changes over time and correlation with patient outcomes require further investigation. Moreover, due to data limitations, we were unable to explore the association between these indicators and early neurological deterioration. The causal relationship between NPAR, NLR, PLR, and functional outcomes in AIS thrombolysis patients remains unclear, emphasizing the need for larger prospective studies to confirm causality and generalize findings to broader populations.

## 5 Conclusion

In summary, NPAR, NLR, PLR, and combined NPAR + NLR levels may predict short-term adverse outcomes in AIS patients receiving intravenous thrombolysis. Among these, NPAR demonstrates superior predictive performance. Using NPAR, NLR, and PLR to predict outcomes in AIS patients undergoing thrombolysis is cost-effective, readily accessible, and holds significant potential for broader application.

## Data Availability

The original contributions presented in the study are included in the article/Supplementary material, further inquiries can be directed to the corresponding author.
